# Splenosis Unmasked: Incidentally Rediscovered in a Patient With Nephrolithiasis and Remote Splenectomy

**DOI:** 10.7759/cureus.112603

**Published:** 2026-07-13

**Authors:** Jason Tasie, Itoye Adatula, Kerolos Hakim Mena, Ahmad Salameh, Monicka Felix

**Affiliations:** 1 Internal Medicine, HCA Florida Trinity Hospital/University of South Florida (USF) Morsani College of Medicine, Trinity, USA

**Keywords:** recurrent nephrolithiasis, splenosis, splenosis after splenectomy, technetium tc-99m, thalassemia

## Abstract

Splenosis is the autotransplantation of splenic tissue that can occur after splenic rupture or splenectomy. It is usually asymptomatic and discovered incidentally. Nephrolithiasis is a common cause of acute flank pain, often diagnosed by non-contrast computed tomography imaging. Splenectomy has been reported as a risk factor for nephrolithiasis in selected hematologic conditions, although its relevance in patients without hematologic disease remains uncertain. A 65-year-old man presented with acute left flank pain. Results of computed tomography imaging of the abdomen confirmed left-sided nephrolithiasis and also revealed multiple well-circumscribed soft tissue nodules in the lower abdomen and pelvis, raising concern for metastatic disease. A detailed history revealed previous splenectomy following an automobile accident at the age of 6 and confirmed splenosis years earlier by technetium scan prior to this admission. The patient’s past medical history of splenectomy and proper history supported the diagnosis of splenosis. This case report highlights the importance of recognizing splenosis as a benign mimic of malignancy on imaging. Thorough history-taking and awareness can prevent unnecessary diagnostic testing and interventions. A history of splenectomy can be a risk factor for nephrolithiasis in individuals with certain hematological diseases.

## Introduction

Splenosis and nephrolithiasis are two distinct medical conditions, and while not directly causally linked, there can be intersections in patient care due to diagnostic considerations or patient-specific risk factors. Furthermore, there is currently limited to no evidence linking splenosis itself with nephrolithiasis.

Splenosis is a rare and benign condition resulting from autotransplantation of splenic tissue after splenic injury or splenectomy. In splenectomies and splenic ruptures, the spleen capsule and parenchyma are torn, consequently releasing small fragments of viable splenic fragments into areas such as the peritoneal cavity [1]. Next, blood or peritoneal fluid distributes the fragments to various locations, commonly peritoneal surfaces, the pelvis, and the diaphragm [2]. Rarely, splenic fragments can reach the brain, thoracic cavity, or subcutaneous tissue [3]. Next, the fragments are implanted on serosal surfaces due to fibrin deposition and local inflammation [4]. Blood vessels begin to form and infiltrate the splenic tissue, keeping the ectopic tissue alive. The fragments then grow, forming splenic nodules usually less than or equal to 3 cm in size. While typically asymptomatic, splenosis can be mistaken for malignancy or lymphadenopathy on imaging [5]. When splenosis occurs in or near the kidney, it can mimic a renal mass, potentially leading to misdiagnosis and unnecessary invasive procedures like partial or total nephrectomy [6]. Diagnostic confirmation of splenosis may be challenging because CT and MRI findings can resemble malignancy. Tc-99m heat-damaged red blood cell scintigraphy, particularly when combined with single-photon emission computed tomography/computed tomography (SPECT/CT), is regarded as the gold standard noninvasive imaging modality because of its high sensitivity and specificity for ectopic splenic tissue, often eliminating the need for biopsy or surgery [5].

Nephrolithiasis is the formation of solid pieces of material (stones) in the kidney from substances in the urine. It is a prevalent condition, and its incidence has been increasing, particularly in younger patients, women, and certain racial groups [7]. Symptoms often arise when stones move from the kidney into the ureter, causing severe pain (renal colic) that may radiate to the lower abdomen or groin. Other symptoms may include nausea, vomiting, blood in the urine (hematuria), frequent urination, painful urination, cloudy or foul-smelling urine, fever, and chills [7]. Treatment options range from conservative management (hydration, pain relievers, medications to help pass stones) to invasive procedures like shockwave lithotripsy (ESWL), ureteroscopy (URS), and percutaneous nephrolithotomy (PCNL), depending on the stone's size, location, and severity of symptoms [7].

Here, we present a case of a patient with known abdominal splenosis who underwent abdominal imaging during a presentation of nephrolithiasis. This case is notable because recurrent nephrolithiasis occurred in a patient with remote splenectomy and a history of splenosis. While splenosis is not a common disease, this report strives to emphasize the importance of including it in differential diagnoses. Diagnostically uncertain cases should be confirmed with nuclear medicine imaging, as this patient underwent before the current presentation. Furthermore, as seen in patients with thalassemia intermedia [8], we investigate whether undergoing a splenectomy is a possible risk factor for recurrent nephrolithiasis [7].

## Case presentation

A 65-year-old man presented to the emergency department with sudden onset of severe left-sided flank pain radiating to the groin. The patient rated the pain 9 out of 10. He denied fever, dysuria, hematuria, and urinary frequency.

The patient’s past medical history included hypertension, hepatic steatosis, and recurrent nephrolithiasis. The patient reported a history of five prior laser lithotripsy procedures for kidney stones, as he had never been able to pass stones spontaneously. Lastly, the patient had a surgical history of splenectomy at the age of 6 because of a motor vehicle accident.

On examination, the patient's vitals included a temperature of 97.3, heart rate of 53, and blood pressure of 192/83. Costovertebral angle tenderness was noted on the left flank. Laboratory tests revealed a white blood cell (WBC) count of 7400 cells×10³/uL, creatinine level of 1.2 mg per dL, potassium of 3.6 mEq/L, and a glucose level of 113. However, the elevated glucose is an incidental finding as it is not a fasting glucose. Urinary analysis revealed clear urine color, trace red blood cells, and a pH of 7.0. Urinary analysis was negative for esterase and protein (Table 1).

**Table 1:** Patient’s complete blood count, basic metabolic panel, and urinalysis.

Lab analysis	Value	Reference
White blood cell	7.4	4.0-10.5 (×10³/uL)
Hemoglobin	15.8	13.7-17.5 (g/dL)
Platelet	263	150-450 (10^3/uL)
Sodium	140	136-145 (mEq/L)
Potassium	3.6	3.5-5.1 (mEq/L)
Creatinine	1.2	0.7-1.3 (mg/dL)
Glucose	113	74-106 (mg/dL)
Calcium	9.5	8.5-10.5(mg/dL)
Urine pH	7.0	4.6-8.0
Urine color	Clear	Clear
Urine esterase	Negative	Negative (cells/uL)
Urine blood	Trace	Negative (cells/hpf)
Urine protein	Negative	Negative (mg/dL)

**Figure 1:**
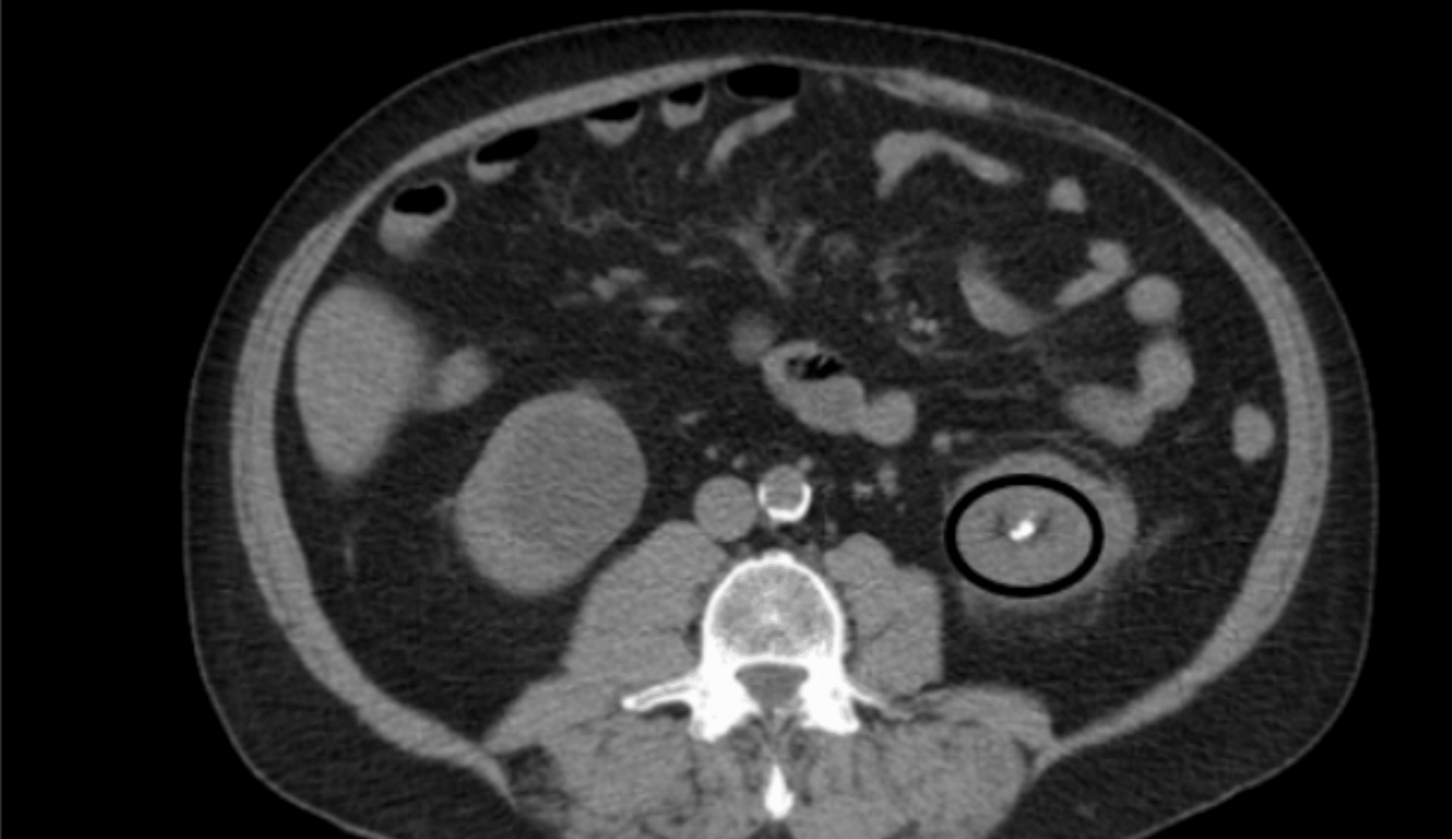
CT scan abdomen axial view: showing a white hyperdense calcified lesion representing a renal stone in left kidney. Mild left hydronephrosis and hydroureter attributed to a 0.3 cm calculus lodged within the distal left ureter.

The results of non-contrast computed tomography (CT) imaging of the abdomen and pelvis revealed mild left-sided hydronephrosis and hydroureter attributed to a 0.3 cm calculus lodged within the distal left ureter (Figure 1).

**Figure 2:**
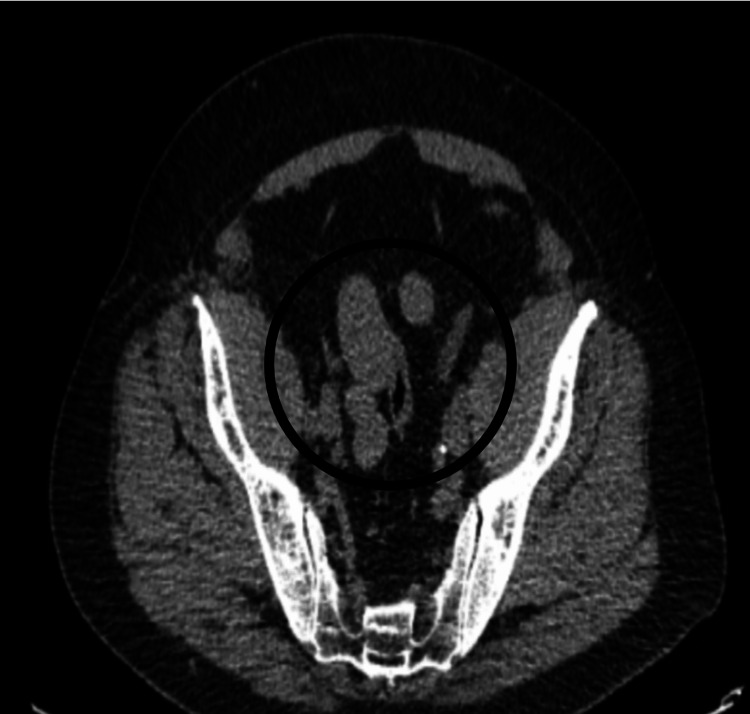
CT scan of the lower abdomen axial view: showing soft tissue masses in the lower abdomen concerning for ectopic splenic tissue. Cluster of soft tissue nodules in the lower abdomen/upper pelvis, some of which are matted together and the largest measuring up to 4.7 cm. Findings are concerning for malignancy, either metastatic disease or lymphoma. Note that splenosis is also possible in this patient given the absence of a spleen in its expected location. Recommend correlation with patient history and consider further workup.

Also found through the same CT scan were several sub-centimeter non-obstructing calculi (the largest measuring 0.5 cm on its longest dimension) at the lower pole of the left kidney. Incidentally, the CT imaging results showed clusters of soft tissue nodules in the lower abdomen and upper pelvis, some of which were matted together, with the largest measuring up to 4.7 cm (Figure 2). Findings were initially concerning for malignancy.

We also noted that the spleen was absent from the abdominal imaging. Given the distribution of the soft tissue nodules, absence of the spleen, and history of splenectomy, splenosis was favored in the differential. When explaining imaging results to the patient, they revealed they are aware of soft tissue nodules, having undergone a heat-damaged technetium-99m-labeled red blood cell scintigraphy years before, which confirmed the diagnosis of splenosis.

The patient was managed conservatively for nephrolithiasis with pain control, including acetaminophen and morphine. The patient was also given normal saline for hydration, along with tamsulosin to relax ureteral and bladder neck smooth muscle via alpha-1 blockade. Urology performed a diagnostic left ureteroscopy, which included placement of a ureteral stent with a string to allow the stones to pass on their own. The patient followed up a week later for stent removal. Since the patient was previously diagnosed with splenosis from prior workup, he was aware that lower abdominal nodules are benign. We recommended that the patient follow up with a primary care physician and his urologist.

## Discussion

Splenosis is an acquired condition in which splenic tissue implants and vascularizes in ectopic locations, typically after splenic rupture [1]. It differs from accessory spleens (congenital) and is often misinterpreted on imaging [9]. Its prevalence is estimated to be up to 67% among patients with a history of traumatic splenectomy [1]. The diagnosis is often incidental, as in this patient’s case. CT imaging results may not distinguish splenosis from malignancy, especially when lesions are multiple or peritoneally scattered. Nuclear medicine techniques, such as a technetium-99 m sulfur colloid scan, offer high specificity in identifying splenic tissue [2]. Nephrolithiasis, on the other hand, is common and easily identifiable by CT. The coexistence of nephrolithiasis and splenosis in this patient highlights the importance of correlating imaging findings with clinical history to avoid misdiagnosis, biopsy, or unnecessary oncologic workup.

While splenosis is usually an incidental imaging finding, a causal association between splenosis and nephrolithiasis has not been shown in the current literature or studies present today. However, an association has been documented in patients with nephrolithiasis and certain underlying hematologic disorders who have had a splenectomy. Specifically, one study suggests that splenectomy may be a risk factor for developing hyperuricemia and nephrolithiasis in patients with certain conditions like thalassemia intermedia [7]. When a patient with splenosis presents with symptoms suggestive of nephrolithiasis (e.g., flank pain and hematuria), careful evaluation is crucial to differentiate between the two conditions and avoid misdiagnosis. Imaging techniques and, in some cases, biopsy or nuclear medicine scintigraphy may be necessary for accurate diagnosis. Thus, a presentation of both splenosis and nephrolithiasis requires a comprehensive approach to accurately diagnose both disorders and effectively manage symptoms and potential complications, while considering the patient’s unique medical history and risk factors.

The retrospective study by Ricchi et al. reported that splenectomy was a major contributor to the formation of nephrolithiasis in patients with thalassemia. For patients with thalassemia intermedia, splenectomy increases the erythrocyte turnover, leading to an increase in uric acid in the blood and consequently increasing the likelihood of nephrolithiasis [7]. While the study did not examine whether the same association is true in patients without thalassemia intermedia, the findings are noteworthy.

This case is reported to highlight how two distinct pathologies may interact to increase risk in a patient with a specific chronic medical condition. This case suggests a possible association warranting further investigation.

Future studies should investigate whether the association between nephrolithiasis and splenosis is also present in populations without thalassemia intermedia, such as our patient. While some genetic factors have been shown to increase a patient's risk for nephrolithiasis [10], further research could investigate whether undergoing a splenectomy is a risk factor for recurrent nephrolithiasis. This could be emphasizing possible prophylaxis or treatments that can prevent the formation of renal stones in individuals with splenectomies or asplenia.

## Conclusions

This case demonstrates the importance of maintaining a broad differential diagnosis when encountering unexpected imaging findings. Splenosis is a benign condition that can present a diagnostic challenge because its imaging appearance may mimic malignancy or other pathological processes. A thorough clinical history, particularly of prior splenic trauma, splenectomy, or history of splenosis, is essential to facilitate accurate diagnosis and avoid unnecessary invasive procedures. When diagnostic uncertainty exists, Tc-99m heat-damaged red blood cell scintigraphy remains a highly sensitive and specific noninvasive modality for confirming ectopic splenic tissue. This case highlights the importance of considering splenosis in the differential diagnosis of incidental abdominal lesions identified during the evaluation of unrelated conditions such as nephrolithiasis. Although an association has been shown between thalassemia Intermedia and nephrolithiasis in patients with a history of splenectomy, this patient had recurrent nephrolithiasis in the setting of prior splenectomy and known splenosis without thalassemia intermedia. As a result, an association cannot be confirmed without further studies. Hence, additional research is needed to determine whether a clinical association exists between splenosis and nephrolithiasis.
